# Dermatofibrosarcoma protuberans with fibrosarcomatous transformation of the abdominal wall: a case report and literature review

**DOI:** 10.1097/RC9.0000000000001211

**Published:** 2026-10-23

**Authors:** Nizar Kardoun, Wael Boujelbene, Wassim Hentati, Amine Chaabouni, Hanen Abida, Salah Boujelben

**Affiliations:** a General Surgery Department, Habib Bourguiba Hospital, University of Sfax, Sfax, Tunisia; b Department of Anesthesiology, Habib Bourguiba Hospital, University of Sfax, Sfax, Tunisia

**Keywords:** case report, dermatofibrosarcoma protuberans, diagnosis, fibrosarcomatous transformation, prognosis, treatment

## Abstract

**Introduction and importance:**

Dermatofibrosarcoma protuberans (DFSP) is a rare, locally aggressive cutaneous sarcoma. Its fibrosarcomatous variant (FS-DFSP) is an uncommon, high-grade subtype associated with increased risks of recurrence and metastasis.

**Case presentation:**

A 43-year-old woman presented with a left iliac fossa skin mass evolving over 20 years, recently characterized by ulceration and bleeding. Computed tomography showed a well-defined anterior abdominal wall mass, initially mimicking a desmoid tumor. Biopsy suggested leiomyosarcoma or FS-DFSP; staging workup was negative. The patient underwent complete *en bloc* resection under general anesthesia. Histopathology confirmed fédération nationale des centres de lutte contre le cancer (FNCLCC) grade 2 FS-DFSP (70% FS component), staged pT2. The immunohistochemical study showed diffuse labeling of tumor cells across both components for CD34, with no staining for smooth muscle actin and caldesmon. Postoperative recovery was uneventful. Due to close margins (3 mm) near the iliac bone, adjuvant radiotherapy was administered (60 Gy, 2 Gy/day, 5 days per week). No recurrence occurred at the 2-year follow-up.

**Clinical discussion:**

FS-DFSP poses diagnostic challenges due to nonspecific clinical and radiological features, requiring histopathological and immunohistochemical confirmation. While complete surgical excision is the cornerstone of treatment, adjuvant radiotherapy helps reduce the risk of local recurrence in cases with close or positive margins.

**Conclusion:**

Managing FS-DFSP requires precise staging, clear margins, and a multidisciplinary consensus, including adjuvant radiotherapy when anatomical constraints limit surgical margins.

## Introduction

Dermatofibrosarcoma protuberans (DFSP), also known as Darier–Ferrand dermatofibrosarcoma, is a rare fibroblastic dermal sarcoma accounting for less than 1% of soft tissue sarcomas^[^1,2^]^. Characterized by slow growth and local tissue infiltration, it carries a high local recurrence risk but low metastatic potential (< 1–2%)^[^3,4^]^. However, fibrosarcomatous transformation (FS-DFSP), seen in 10–15% of cases, represents a more aggressive variant with higher rates of recurrence (30–50%) and systemic metastasis (10–15%)^[^5^]^. DFSP typically affects adults aged 40–43 years^[^1^]^.

HIGHLIGHTS

- A rare case of dermatofibrosarcoma protuberans with a 20-year history of slow progression.
- Histopathology confirmed a high-grade fibrosarcomatous component comprising 70% of the tumor.
- Complete resection was achieved with close margins, prompting adjuvant radiotherapy.
- No local recurrence or distant metastasis was observed at the 2-year follow-up.

We report an unusual case of FS-DFSP in a 43-year-old woman with a 20-year history involving the left iliac fossa. Histopathology revealed a 70% FS component. Due to the proximity of the iliac bone, narrow surgical margins were achieved, necessitating adjuvant radiotherapy. The patient remains disease-free at 2 years of follow-up. This case highlights the management of aggressive FS-DFSP under anatomical margin constraints. This report adheres to the SCARE 2025 guidelines^[^6^]^.

## Case report

A 43-year-old non-smoking Tunisian woman with no significant medical or surgical history presented with a 20-year history of a slowly growing, asymptomatic left iliac fossa skin lesion. Over the preceding 2 months, the mass exhibited rapid progression with ulceration and bleeding. The patient was hemodynamically stable with a body mass index of 26 kg/m^2^. Physical examination revealed a 10 cm, mobile, ulcerated anterior abdominal wall mass with minimal active bleeding (Fig. 1). No regional or distant lymphadenopathy was detected. Routine laboratory investigations – including complete blood count, renal panel, and coagulation profile – were unremarkable.

**Figure 1.**
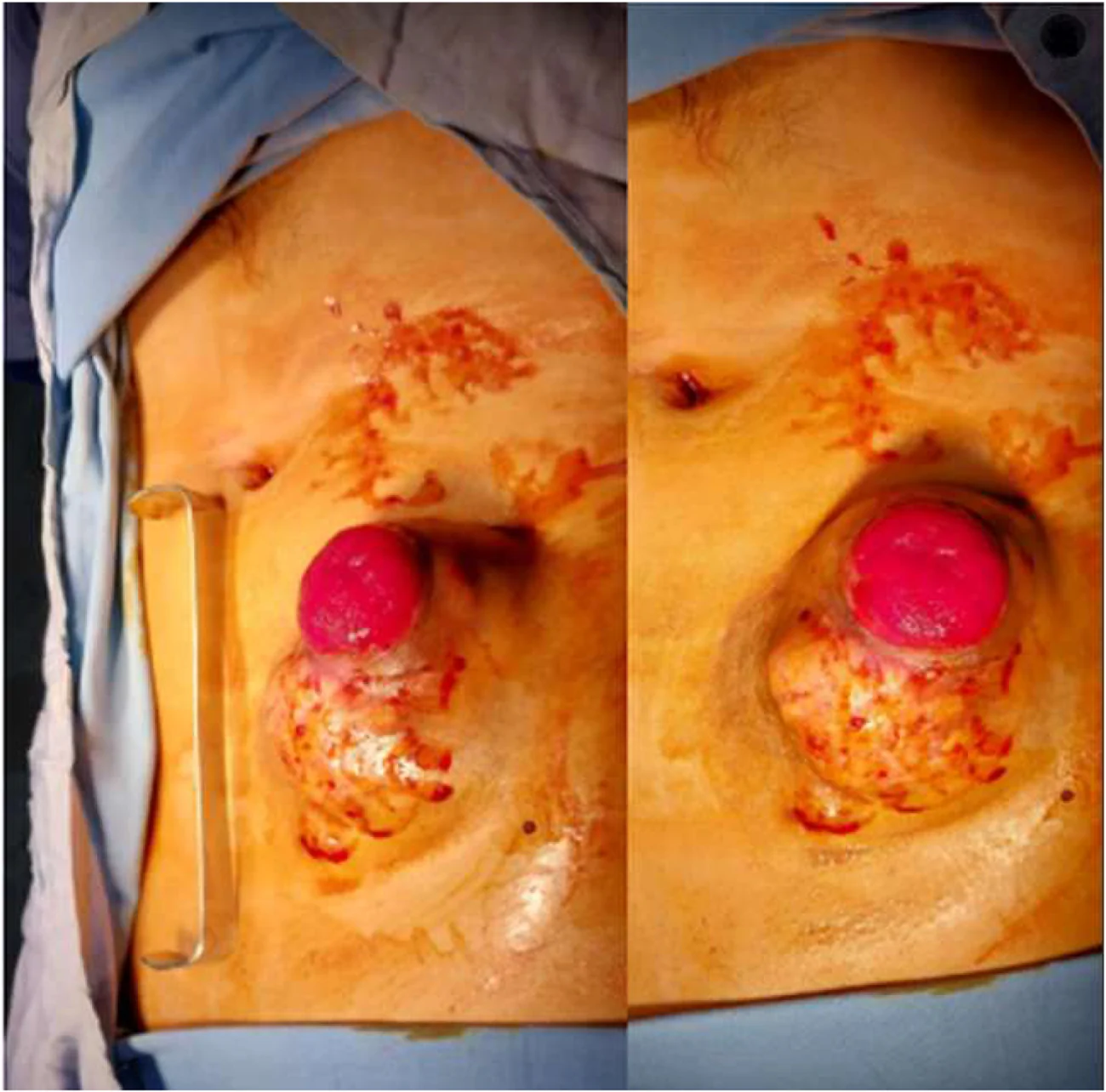
Clinical appearance of an ulcerated left iliac fossa mass with spontaneous bleeding, suggestive of locally advanced dermatofibrosarcoma protuberans.

Abdominopelvic computed tomography (CT) revealed a well-circumscribed, macronodular soft-tissue mass (50 × 80 × 90 mm) in the left anterior abdominal wall with homogeneous contrast enhancement and clear planes with the rectus abdominis muscle (Fig. 2), initially raising suspicion for a desmoid tumor. A punch biopsy demonstrated a spindle-cell proliferation concerning for leiomyosarcoma or FS-DFSP; immunohistochemistry was not performed on the biopsy specimen. Staging chest-abdomen-pelvis CT showed no distant metastasis.

**Figure 2.**
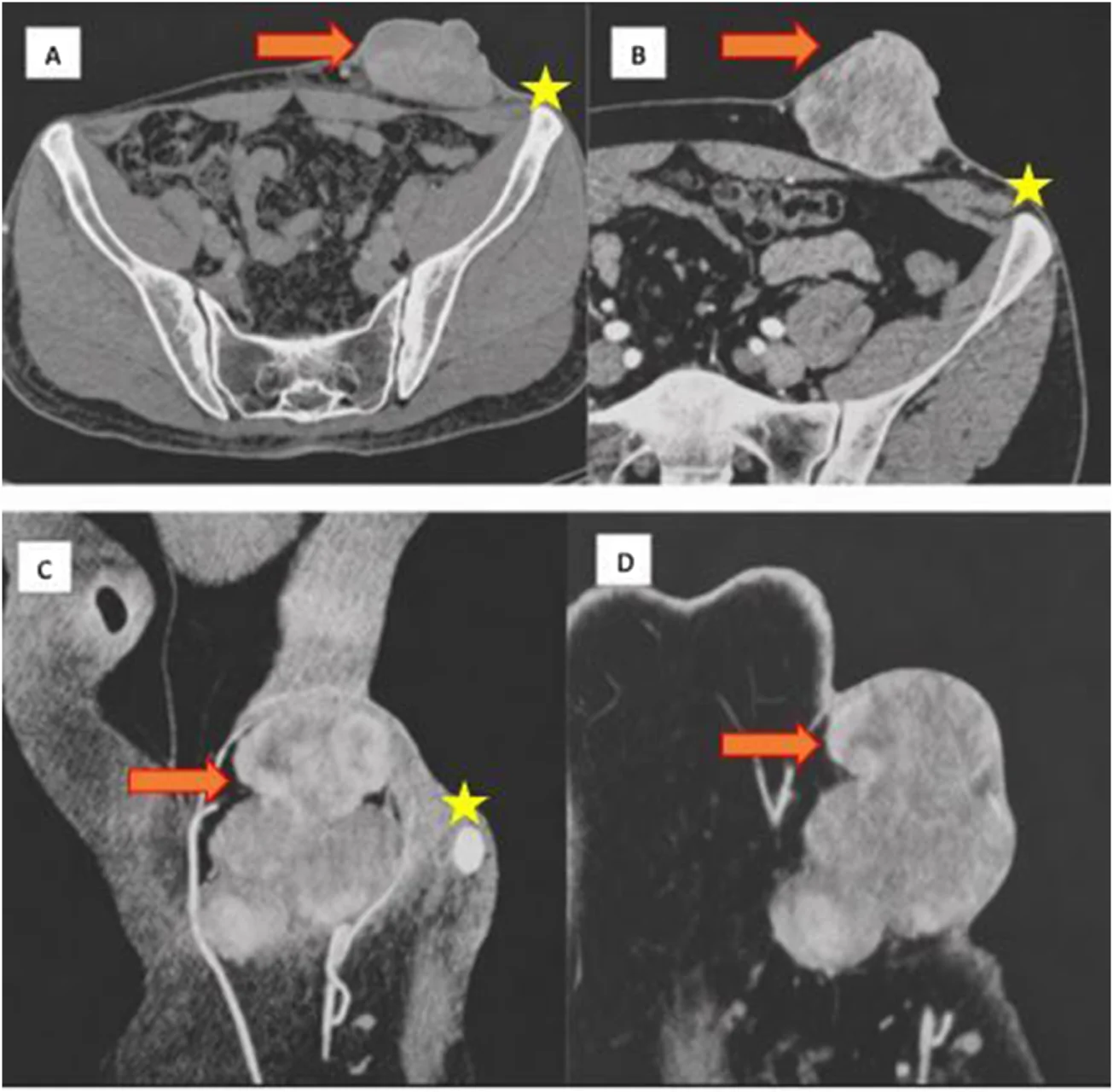
Abdominopelvic CT scan showing a large, well-circumscribed, macro-nodular soft-tissue mass (arrow) in the left anterior abdominal wall with homogeneous contrast enhancement. (A) Axial CT scan showing a well-defined, exophytic soft-tissue mass (arrow) arising from the left anterior abdominal wall, in proximity to the underlying iliac bone (*). (B) Axial contrast-enhanced image demonstrating homogeneous enhancement of the lesion (arrow), with a preserved cleavage plane between the lesion and adjacent muscular structures, in proximity to the underlying iliac bone (*). (C) Coronal reconstruction of the tumor (arrow). (D) Sagittal reconstruction.

Following multidisciplinary team discussion, the patient underwent *en bloc* surgical resection under general anesthesia in a university hospital setting, led by a professor of general surgery (Fig. 3). Wide local excision (WLE) achieved 2 cm lateral margins, except adjacent to the iliac bone, where anatomical constraints limited the margin to 1 cm. The tumor spared the rectus abdominis muscle and aponeurosis. The postoperative wound measured approximately 9 × 11 cm, and intraoperative blood loss was minimal (less than 300 mL), with no need for blood transfusion. Following limited subcutaneous dissection, primary layered closure was performed. Total operative time was 90 minutes. Postoperative recovery was uneventful, and the patient was discharged on postoperative day 2.

**Figure 3.**
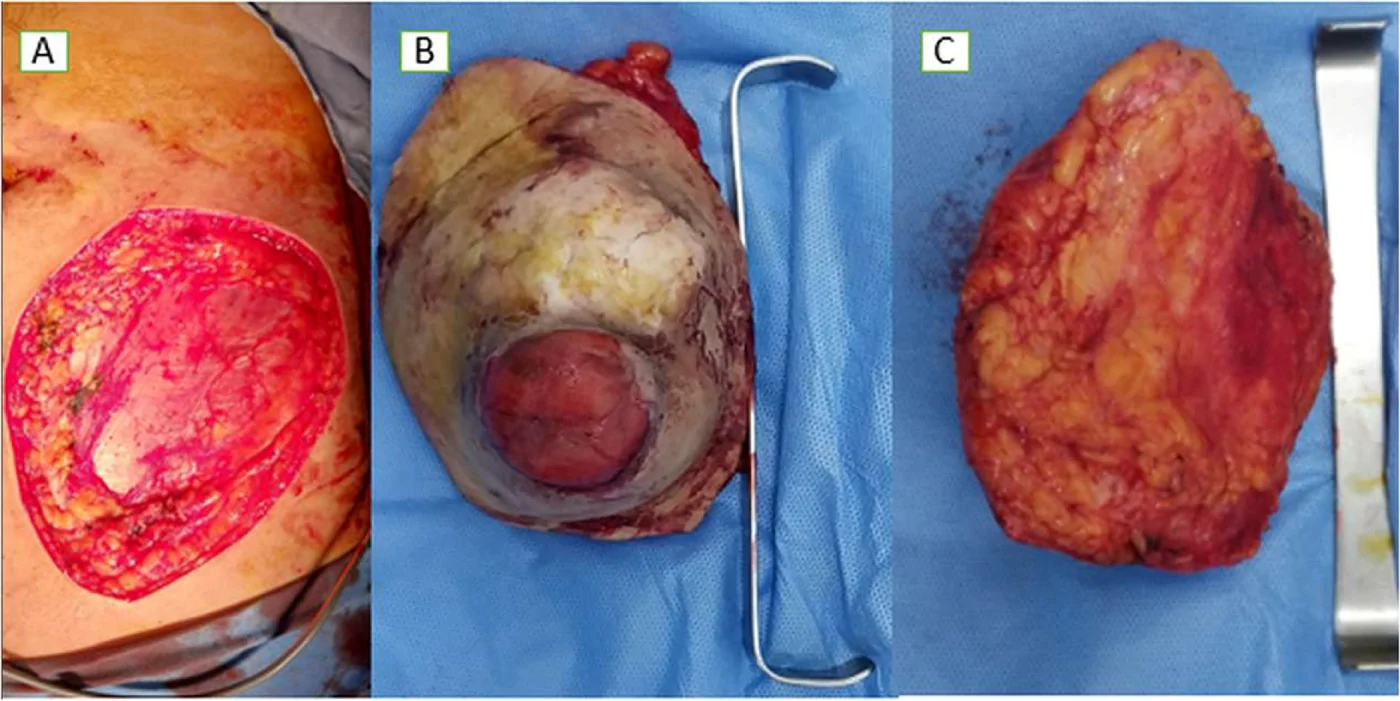
Surgical specimens following complete tumor excision: (A) Intraoperative view following tumor excision. (B) *En bloc* surgical specimen with overlying ulcerated skin; (C) Gross specimen of the resected tumor.

Histopathology confirmed a 9 × 7 cm fédération nationale des centres de lutte contre le cancer (FNCLCC) grade 2 (2 + 3 + 0) FS-DFSP (70% FS component), staged as pT2 (AJCC 8th edition). The tumor exhibited a biphasic pattern: a peripheral conventional DFSP component (30%) with uniform, low-grade spindle cells infiltrating subcutaneous fat in a storiform architecture and rare mitoses, and a central FS transformation (70%) with hypercellular, atypical fusiform cells in a herringbone pattern, 14 mitoses/mm^2^, and focal edematous-hyaline changes, but no geographic necrosis. No lymphovascular or perineural invasion was identified. Immunohistochemistry showed diffuse CD34 positivity in both components, with smooth muscle actin and caldesmon both negative. The closest microscopic margin was 3 mm near the iliac bone. Following multidisciplinary discussion, and given the close margin and the inability to re-excise the underlying bone in the absence of gross residual disease, adjuvant external-beam radiotherapy (60 Gy in 2 Gy fractions) was administered 2 months postoperatively. At 2-year follow-up, clinical and radiological surveillance every 6 months showed no disease recurrence. Imaging consisted of alternating thoraco-abdomino-pelvic CT and abdominal magnetic resonance imaging (MRI), with chest CT every 6 months.

## Discussion

We report on a 43-year-old woman with a long-standing, ulcerated left iliac fossa DFSP with a 70% FS component. Following surgical resection with close bone margins, adjuvant radiotherapy resulted in disease-free survival at 2 years. This case highlights the clinical, radiological, and histopathological features of FS-DFSP, as well as its therapeutic management under anatomical constraints. Below, we discuss these findings in light of the current literature to outline optimal diagnostic and treatment strategies.

### Clinical characteristics

DFSP is a low-grade cutaneous sarcoma originating in the dermis, with an infiltrative growth pattern extending into subcutaneous tissues, fascia, and bone^[^7^]^. Typically involving the trunk, it presents as a slow-growing, flesh-colored to yellow-brown indurated plaque or nodular lesion^[^8^]^. Early-stage tumors often remain indolent and non-protuberant for years (averaging 7 years), frequently delaying diagnosis due to clinical similarity with benign conditions^[^7^]^. However, rapid enlargement or morphological changes suggest a higher-grade FS transformation^[^8^]^. In these aggressive variants, histopathological evaluation is mandatory before surgical intervention to guide optimal management^[^7^]^. Our 43-year-old patient presented with a 20-year history of indolent disease preceding rapid tumor growth, ulceration, and bleeding – a course characteristic of high-grade transformation in abdominal-wall FS-DFSP.

### Imaging evaluation

Ultrasound is valuable for the initial evaluation of DFSP, typically revealing a well-defined hypoechoic or heterogeneous subcutaneous mass with a characteristic honeycomb pattern and increased Doppler vascularity^[^7^]^. High-frequency probes (10–20 MHz) accurately assess tumor depth and fascial involvement^[^7^]^.

CT shows a nodular subcutaneous lesion with soft-tissue attenuation and contrast enhancement^[^9^]^. In tumors larger than 5 cm, non-enhancing areas indicate necrosis or degeneration. CT also serves to screen for distant metastases^[^1^]^.

MRI is crucial for surgical planning and postoperative surveillance, particularly in large or recurrent lesions, achieving 67% sensitivity and 100% specificity^[^1,7,10^]^. DFSP appears iso- to hypointense to muscle on T1-weighted sequences and intermediate to hyperintense on T2-weighted or fat-suppressed images^[^7^]^. In the postoperative setting, MRI reliably detects recurrence, typically appearing as well-defined, strongly enhancing nodular lesions^[^1,7^]^.

Although not routine, positron-emission tomography /CT helps evaluate treatment response and staging in high-grade or FS variants, where elevated metabolic activity reflects aggressive behavior^[^1,7^]^.

In our patient, initial abdominopelvic CT revealed a 5 × 8 × 9 cm mass mimicking a desmoid tumor. Preoperative staging with thoraco-abdomino-pelvic CT effectively excluded distant metastases prior to radical resection; MRI was omitted due to financial constraints.

### Pathologic diagnosis

Definitive diagnosis relies on histopathological examination, typically obtained via incisional or excisional biopsy; fine-needle aspiration is avoided due to limited reliability^[^7^]^.

Histologically, classic DFSP is characterized by a proliferation of uniform spindle cells arranged in a distinctive storiform pattern, with minimal cytological atypia and low mitotic activity. These cells characteristically show strong CD34 immunoreactivity^[^4^]^.

Although several variants exist (including Bednář tumor, myxoid, and giant-cell fibroblastoma), FS-DFSP represents a distinct, aggressive subtype^[^4^]^. Microscopically, FS-DFSP areas exhibit increased nuclear atypia, elevated mitotic activity, and a transition from the classic storiform architecture to a herringbone growth pattern^[^4^]^. Notably, CD34 expression is frequently reduced or entirely lost in these FS regions^[^4^]^.

Histopathology in our case confirmed a biphasic tumor with a predominant (70%) FS component, which the literature strongly correlates with increased aggressiveness and risk of local recurrence. Notably, while FS-DFSP typically exhibits loss of CD34 expression, tumor cells in our case retained diffuse CD34 positivity across both conventional and FS components.

### Clinical staging system

DFSP lacks a definitive staging system^[^4^]^. While some authors use the AJCC soft tissue sarcoma protocol^[^11^]^, others rely on simpler classifications: The National Comprehensive Cancer Network defines localized disease as stage I, regional as stage II, and distant metastasis as stage III^[^3,12^]^.

Similarly, the 2018 German guidelines classify a localized tumor as stage I, regional lymph node involvement as stage II, and distant disease as stage III^[^13^]^.

Imaging techniques play a role in DFSP staging^[^13^]^.

Based on the 8th edition of the AJCC/pathological tumor–node–metastasis classification, our patient was staged as T2. However, the presence of FS transformation warrants closer surveillance due to its higher metastatic potential compared with that of conventional DFSP.

### Treatment

Primary management of DFSP aims for complete surgical resection during the initial intervention^[^12^]^. Due to the tumor’s irregular, infiltrative, finger-like extensions, complete excision is challenging^[^12^]^. Consequently, margin-controlled techniques – such as Mohs micrographic surgery or Peripheral and Deep En Face Margin Assessment – are preferred over WLE to minimize recurrence^[^12^]^. When critical neurovascular or osseous structures limit the evaluation of deep margins, a hybrid approach combining margin control and conventional excision may be guided by multidisciplinary discussion^[^12^]^.

Recommended margins vary: 1 cm is sufficient with three-dimensional micrographic control (e.g., Mohs or Breuninger methods), whereas traditional histological assessment requires margins of at least 2 cm^[^13^]^. If initial margins are positive, re-resection is mandatory whenever feasible^[^12^]^. For FS variants, cases must be reviewed by a specialized soft-tissue sarcoma board; management requires complete excision via 3D margin-controlled techniques or WLE with 3 cm margins to reduce recurrence and metastatic risks^[^8^]^.

In our case, WLE achieved a 2 cm lateral margin, but anatomical proximity to the iliac bone constrained the surgical margin to 1 cm macroscopically and 3 mm microscopically. Despite avoiding bone resection, complete macro- and microscopic clearance was achieved.

Radiotherapy is primarily used as an adjuvant therapy after surgery rather than as a definitive treatment^[^12^]^. It enhances local control and reduces recurrence risk, particularly for close or positive margins^[^12^]^. Postoperative radiotherapy can significantly reduce the risk of recurrence. Evidence from a meta-analysis including 12 studies indicated a pooled recurrence rate of 11.74% after adjuvant radiotherapy, with higher recurrence in patients with positive or close margins (14.23%) and no recurrences observed in those with negative margins^[^14^]^. Although the difference between surgery alone and adjuvant radiotherapy lacks statistical significance, a trend toward lower recurrence persists^[^14^]^. Current recommendations suggest considering radiotherapy when margin-controlled techniques (e.g., Mohs surgery) are omitted, surgical margins are narrow, or further resection is infeasible^[^12^]^.

Given the narrow microscopic deep margin (3 mm near the iliac bone) and the high-grade FS histology, adjuvant radiotherapy (60 Gy) was delivered to optimize local control, resulting in no evidence of disease at 24 months.

Systemic treatment of DFSP relies on targeted therapy rather than conventional chemotherapy^[^12^]^. The characteristic *t*(17;22) chromosomal translocation leads to platelet-derived growth factor receptor beta overexpression, a key therapeutic target^[^7^]^. Consequently, imatinib mesylate is approved for unresectable, recurrent, or metastatic disease, and serves in neoadjuvant, adjuvant, or palliative settings^[^12^]^. Neoadjuvant imatinib is recommended for borderline resectable tumors or when negative surgical margins would cause unacceptable functional or cosmetic morbidity^[^12^]^. While clinical responses are primarily partial, the predictive value of the *COL1A1-PDGFB* fusion gene remains under investigation^[^7,12^]^. Systemic therapy was not indicated in our patient, as primary complete surgical resection was feasible and staging ruled out distant disease.

### Prognosis and surveillance

DFSP generally has a favorable prognosis, with reported 5-year recurrence-free survival of 86% and 10-year recurrence-free survival of 76%^[^15^]^. However, advanced age, high mitotic rates, increased cellularity, and positive margins correlate with worse outcomes^[^1^]^. Local recurrence remains the primary challenge, ranging from 20 to 50%, with a median onset of 32–38 months post-excision^[^15^]^.

Consequently, long-term surveillance is essential. Guidelines recommend clinical examinations every 6–12 months, extended to 10 years or more, focusing on the primary scar site^[^3,4,12,13^]^. Patient education on self-examination is critical^[^12^]^. While routine imaging is not systematically required, MRI should be considered for high-risk features like FS-DFSP^[^4,12^]^. Recurrences are managed with re-resection and adjuvant radiotherapy^[^12^]^, whereas unresectable or metastatic disease requires imatinib therapy and multidisciplinary management^[^12^]^.

Given the high-risk profile (70% FS component, 3 mm deep margin), our patient remains under strict clinical and radiological surveillance every 6 months and is disease-free at 2 years post-treatment.

## Conclusion

While conventional DFSP rarely metastasizes, FS-DFSP significantly increases the risk of local recurrence and systemic spread. This case underscores the importance of precise histological quantification of the fibrosarcomatous component and careful margin evaluation adjacent to complex bony structures. Rather than routine application, our multidisciplinary team selectively utilized adjuvant radiotherapy to address a close 3 mm-deep margin, achieving complete disease control at 2 years. Given the aggressive nature of FS-DFSP, structured long-term surveillance combining local imaging and chest CT remains essential for early detection of late recurrence or metastasis.

## Patient perspective

Because of 20 years of painless growth, the patient delayed seeking care until rapid enlargement and bleeding occurred over the course of 2 months. She recovered quickly after complete surgical excision, which spared deep structures, and adjuvant radiotherapy was deemed necessary given the close margin. Regular 6-month clinical and radiological surveillance has provided sustained reassurance, with full functional recovery and no recurrence at 2 years.

## Data Availability

The datasets generated and analyzed during the current case report are not publicly available due to patient privacy and confidentiality restrictions. De-identified data and clinical documentation are available from the corresponding author upon reasonable request.
